# Conservative management of cervical intraepithelial neoplasia grade 2 and immunohistochemical staining of p16 and Ki-67: Data from a Brazilian institution

**DOI:** 10.1371/journal.pone.0342001

**Published:** 2026-02-25

**Authors:** Amanda Leal Ferreira, Yara Lúcia Mendes Furtado de Melo, Elyzabeth Avvad Portari, Dione Corrêa Araújo Dock, Nilma Valéria Ferreira Caldeira, Saint Clair dos Santos Gomes Júnior, Fabio Bastos Russomano, Cecília Vianna de Andrade

**Affiliations:** 1 Laboratory of Pathology and Cytopathology, Fernandes Figueira National Institute of Women, Child, and Adolescent Health of Fiocruz, Rio de Janeiro, Rio de Janeiro, Brazil; 2 Cervical Pathology, Gynecology Institute of the Federal University of Rio de Janeiro, Rio de Janeiro, Rio de Janeiro, Brazil; 3 Clinical Research Unit, Fernandes Figueira National Institute of Women, Child, and Adolescent Health of Fiocruz, Rio de Janeiro, Rio de Janeiro, Brazil; 4 Clinical and Surgical Care of Women’s Area, Fernandes Figueira National Institute of Women, Child, and Adolescent Health of Fiocruz, Rio de Janeiro, Rio de Janeiro, Brazil; Nelson Mandela African Institute of Science and Technology, TANZANIA, UNITED REPUBLIC OF

## Abstract

**Introduction:**

Cervical intraepithelial neoplasia grade 2 (CIN2) includes both transient HPV infections and precursor lesions of cervical cancer. Conservative management aims to avoid overtreatment, especially in reproductive-aged women.

**Objective:**

To describe the clinical outcomes and time to lesion regression among conservatively managed, biopsy-confirmed CIN2 cases, and to explore whether baseline characteristics, including p16 and Ki-67, were associated with lesion regression.

**Materials and methods:**

An observational cohort study was conducted in patients with CIN2, as determined by cervical biopsy. Participants were followed up with cytology and colposcopy every 6 months, and outcomes were categorized as regression or no regression. At least two pathologists evaluated p16 immunohistochemical staining according to LAST criteria. Ki-67 staining was considered positive when at least 50% of cells in the lesion were stained, as determined by digital quantification. Bivariate analysis and survival curve analyses were conducted, with significant associations defined as p-values <0.05.

**Results:**

Fifty patients with a mean age of 30.5 years were included in the study; 76% (38/50) showed regression. The median time to regression was 357 days (SE = 61.4 days; 95% CI: 236.6–477.4 days). The baseline characteristics, including p16 and Ki-67, were not statistically associated with regression, likely due to the small sample size.

**Conclusion:**

CIN2 regression occurred in 76% of cases, with median times of approximately 12 months for regression and 32 months for progression. No statistically significant associations were detected between baseline characteristics, including biomarkers, and lesion regression in this exploratory study.

## Introduction

Cervical cancer constitutes a public health problem worldwide, representing the fourth most common cancer type and one of the leading causes of cancer-related deaths in women [1]. Cervical cancer typically develops through a long precancerous phase represented by cervical intraepithelial neoplasia (CIN). While CIN 1 is considered a low-grade lesion (LSIL) without a precursor potential, CIN2 and CIN 3 are regarded as high-grade lesions related to a higher risk for progression to cancer [2–4].

Some studies have consistently shown that most CIN2 lesions regress spontaneously, particularly in women younger than 30. A meta-analysis and recent cohort studies advocate conservative management in selected cases of CIN2, emphasizing personalized care that avoids overtreatment while ensuring oncologic safety [5–9]. This is especially relevant because excisional treatment has been linked to adverse obstetric outcomes [10–14]. Therefore, avoiding unnecessary treatment is particularly important in reproductive-aged women. This discussion is particularly relevant in Brazil and other countries, where guidelines recommend excision for CIN2 based only on patients’ age [15], while maternal age is increasing [16]. The proportion of mothers aged 30 years or older increased from 23.7% in 2000 [17] to 37.7% of live births in 2020 in Brazil [18].

CIN2 is a heterogeneous diagnosis that encompasses both transient HPV-related changes and true precursor lesions. Moreover, the diagnosis of CIN2 is characterized by significant interobserver variability [19–21]. To address this issue, the Lower Anogenital Squamous Terminology (LAST) guidelines recommend using p16 immunohistochemistry to refine CIN2 diagnoses, stratifying lesions into high-grade or low-grade based on p16 staining patterns. This distinction has prognostic implications, as p16-positive CIN2 lesions may be less likely to regress [4]. p16 is a tumor suppressor protein that is consistently overexpressed in high-risk HPV (hr-HPV) associated lesions, correlating with diagnostic reproducibility, diagnostic accuracy, and disease progression [22–30]. Some studies have demonstrated its usefulness in managing CIN2 due to its high negative predictive value; however, p16 positivity alone is not sufficient to indicate excisional treatment [31,32].

Ki-67 is a nuclear proliferation marker that has also been associated with CIN grade and lesion progression [24,27]. However, its prognostic value in guiding treatment versus surveillance remains controversial [32]. There is no consensus on the cutoff point for positive Ki-67 expression, but the 50% cutoff has been associated with CIN 3 or carcinoma in previous studies [24,27].

Therefore, this study aimed to describe the clinical outcomes and time-to-event patterns of conservatively managed, biopsy-confirmed CIN2 cases in a Brazilian public healthcare setting and to explore whether selected baseline characteristics—p16, Ki-67, age, and referral cytology—were associated with lesion regression.

## Materials and methods

### Study design and setting

This was a bidirectional cohort study, including both retrospectively identified and prospectively enrolled patients, at a reference center for cervical uterine pathology and colposcopy within the Brazilian Unified Health System (SUS) in Rio de Janeiro, Brazil. Cases were identified from the institutional database (2006–2018) and from consecutive referrals to the colposcopy clinic during the study period (2018–2022).

All patients were referred to colposcopy due to abnormal cervical cytology originating from primary care units within the same geographic region. Diagnostic criteria, colposcopy procedures, immunohistochemical protocols, histopathological review, and outcome definitions remained unchanged throughout the study period, ensuring consistency in data collection and clinical management.

### Cohort definition

Given that all patients originated from the same source population and were investigated, managed, reviewed, and followed using identical clinical and pathological protocols, all cases were analyzed as a single cohort.

### Sample size

All cases that met the inclusion/exclusion criteria during the study period determined the sample size. Although a larger sample size had been initially planned, the final cohort reflects the cases available for analysis within the predefined study period.

### Inclusion criteria

Patients with confirmed biopsy diagnosis of CIN2 on H&E, independently reviewed by two pathologists, who had not undergone treatment at the time of diagnosis, presented with a fully visible SCJ on colposcopy (transformation zones types 1 or 2), and had no previous history of pre-invasive or invasive cervical disease.

### Exclusion criteria

Patients living with Human Immunodeficiency Virus (HIV) or other immunodeficiency conditions, pregnant women, cases immediately treated, cases in which the cervical biopsy was considered excisional (defined as cases in which the entire lesion was intentionally removed during the procedure), cases of biopsy failure were excluded from the study (defined as cases where a more severe diagnosis was made at the immediately subsequent visit), and cases with no follow-up proposed (defined as cases in which immediate treatment was proposed but patient did not return for treatment).

### Follow-up

All women included in this study were prospectively followed according to a standardized conservative management protocol, including semiannual cytology and colposcopy, until treatment or discharge from follow-up. Excisional treatment was undertaken if persistent lesions were evident after 24 months of follow-up or if progression occurred at any point during the observation period.

Clinical follow-up procedures and outcomes assessment were identical for all women, regardless of the period of inclusion. For women enrolled earlier in the study period (before July 2018), baseline and follow-up information were retrieved from medical records; for those enrolled later (from July 2018), the data were collected prospectively during scheduled visits. Importantly, in both situations, the decision to treat or not was based on cytological and colposcopic evaluation, following the same institutional protocol.

Women who, despite not presenting with cytological or colposcopic evidence of progression, opted for treatment during follow-up were also included and contributed to the total follow-up time. This study was concluded on May 31, 2024.

Loss to follow-up was defined as the absence of, or incomplete, follow-up examination after the initial biopsy, preventing a proper determination of their outcomes. These losses were attributed to social factors and were unrelated to the outcomes.

### Follow-up time

For each participant, follow-up began on the date of the diagnostic (index) biopsy, which confirmed CIN2. Participants were followed until the earliest of: (1) date of excisional treatment, (2) date of histologically confirmed progression (CIN3 or carcinoma), (3) date of last clinical contact (right-censoring), (4) date of first documented regression (histological regression to ≤CIN1 or cytology/colposcopy criteria consistent with regression as defined ahead. In this case, the date of the first documented regression was used to define the time to regression. However, patients continued to be monitored for at least 2 years of follow-up. If evidence of persistence or progression was identified during this period, the patient was reclassified as demonstrating no regression, and the date on which progression or persistence was observed was recorded for analysis purposes. Event dates correspond to the date of diagnostic confirmation (histology or cytology/colposcopy). Because events were ascertained at scheduled semiannual visits, exact times of biological change may fall between visits.

### Clinical outcomes

This analysis classified outcomes as regression, persistence, or progression at the end of the patient’s follow-up period, regardless of whether this was due to excisional treatment, patient dropout, discharge, or study period termination.

Regression cases were those with histological results of ≤CIN 1 in the specimen after the initial biopsy or, in the absence of histological data, cytological examinations classified as a normal, inflammatory, low-grade squamous intraepithelial lesion (LSIL) or atypical squamous cells of undetermined significance (ASC-US), and colposcopy with no or minor abnormal findings [33]. Complete regression was defined as the return of all exams performed during follow-up to normal (histopathological or cytological report negative for malignancy or intraepithelial lesion, colposcopy with no abnormal findings, and fully visible SCJ).

Persistence was defined as a histologic diagnosis of CIN2 during follow-up.

The diagnosis of CIN 3 or invasive carcinoma during follow-up was classified as progression. For the analysis, persistence and progression were grouped into ≥ CIN2 (no regression). All included cases were reviewed by the most experienced colposcopist in the service to identify biopsy failures. When the lesion aspect was worsened, progression was considered.

In cases of divergent diagnoses (cytopathological, histopathological, or colposcopic) on the same date, the histopathological diagnosis or the most severe (between cytology and colposcopy) was considered the outcome.

### Colposcopy

Data from colposcopies prior to the initial biopsy and follow-up colposcopies were collected from specific forms and confirmed or completed from patient records. The previous colposcopy was considered the examination performed before the biopsy with a diagnosis of CIN2. Follow-up colposcopies were performed semiannually by experienced colposcopists at the cervical pathology unit. Colposcopy results were classified as normal findings, minor or major abnormal findings, or suggestive of invasion.

### Cytological and histopathological results

Results were obtained from medical records based on cytology reports from different SUS primary care centers. Follow-up cytology was performed by the same cytopathologist from the cervical pathology reference center in RJ, and results were collected from reports attached to medical records. Histopathological results of the biopsies were confirmed by at least two pathologists, who were blinded to the clinical outcome. Histopathological results from excisional treatment were obtained from reports prepared by pathologists at the same cervical pathology reference center.

### Immunohistochemistry

Archived formalin-fixed, paraffin-embedded histological blocks were retrieved when available and used for immunohistochemical analyses. All immunohistochemistry reactions were performed in a single laboratory, as part of this study, following the same protocol.

Immunohistochemical expression of p16 was analyzed using a pre-diluted mouse monoclonal antibody anti-p16 (dilution 1:30; CINtec p16 Histology, Ventana Medical Systems, Tucson, USA) and a monoclonal anti-Ki-67 antibody (1:200 dilution; clone SP6, Cell Marque, Rocklin, USA, using the Max Polymer Detection System, BOND-MAX, Leica, Melbourne, Australia) using an automated system. As a positive control, TMA slides positive for p16 and Ki-67 were prepared according to the method developed by Pires et al. [34]. For the negative control, the primary antibody was replaced by phosphate-buffered saline.

### Evaluation of Immunohistochemical staining

At least two experienced pathologists classified p16 immunohistochemical staining according to the LAST recommendations: strong, diffuse staining in at least one-third of the epithelium was considered positive, whereas focal or multifocal weak or absent staining was considered negative. Consensus diagnosis among pathologists was considered for analysis. In case of disagreement, samples were re-evaluated together to define the classification of p16 immunohistochemical staining.

Ki-67 staining was considered positive when more than 50% of cells in the lesion were stained, as determined by digital quantification. Slides were digitized using the Aperio ScanScope CS5 scanner (Leica, Vista, USA). Ki-67 expression was quantified using Aperio ImageScope software, version 11 (Leica, Vista, USA), using the IHC Nuclear algorithm, version 1 (Leica, Vista, USA), with the pathologist demarcating the quantification area.

### Statistical analysis

Baseline characteristics are presented as mean ± standard deviation (SD) or as n (%). Age was dichotomized at 30 years, based on evidence that CIN2 regression rates are higher in women aged ≤30 years [5].

Bivariate associations between baseline variables and lesion regression were examined using the chi-square test or Fisher’s exact test. Crude odds ratios (OR) with 95% confidence intervals (CI) were reported. For variables with sparse data or zero cells, odds ratios were not estimated due to instability of the estimates.

Time-to-event analyses were performed using Kaplan-Meier curves to estimate the cumulative probability and median time to lesion regression and progression, with corresponding standard errors and 95% confidence intervals. Given the limited number of events and substantial censoring, multivariable time-to-event regression models were not performed. A two-sided p-value ≤ 0.05 was considered statistically significant. All analyses were conducted using SPSS version 20.0.

In addition, a sensitivity analysis was conducted to assess potential differences in outcome distribution and biomarker status between patients included retrospectively from the institutional database and those enrolled prospectively.

### Ethical considerations

The institution’s Research Ethics Committee approved the study under protocol number CAAE 76241617.4.0000.5269. Patients still under active surveillance provided consent at study initiation or at entry for prospectively enrolled cases. Patients who had already been discharged from follow-up had their consent waived.

The data was accessed between 18 July 2018 and 31 May 2022, and only one of the authors had access to information that could identify individual participants during data collection. The colposcopists were responsible for treatment decisions based on patient history, cytology results, colposcopic impression, and patient preferences.

## Results

**Fig 1:**
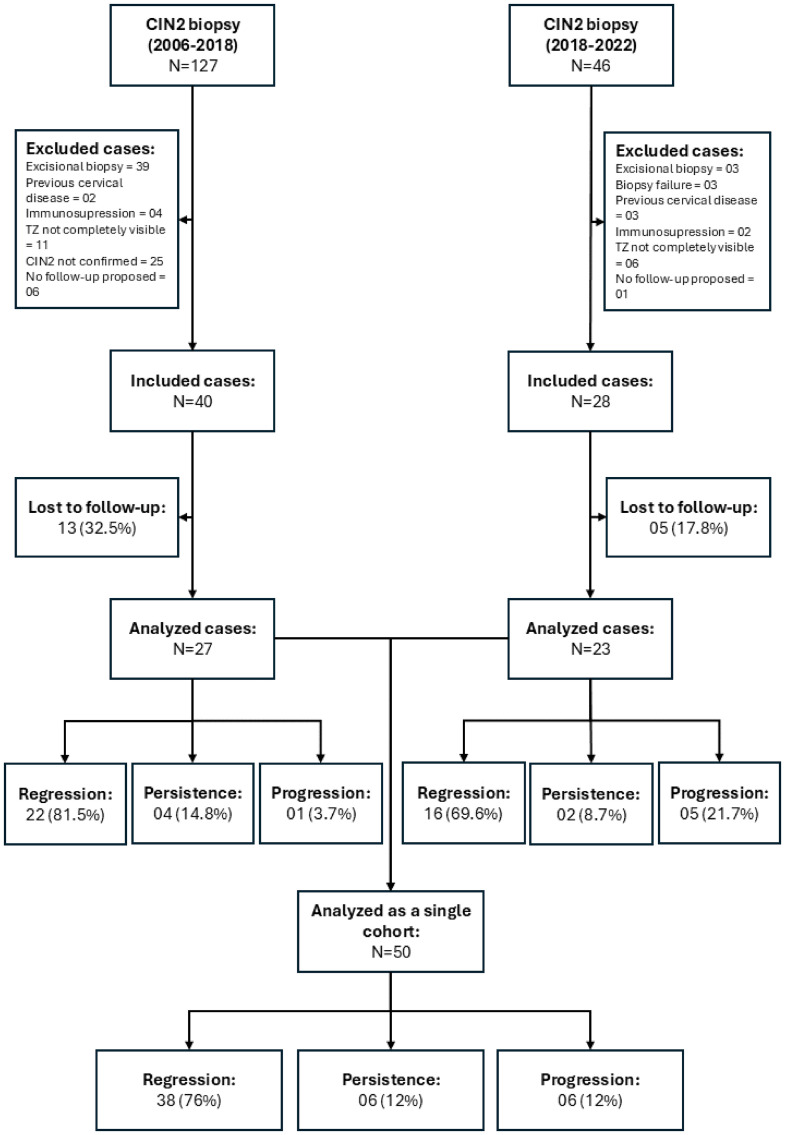
Flowchart of patient inclusion in the study and outcomes. CIN: Cervical Intraepithelial Neoplasia; ZT: Transformation Zone; no follow-up proposal: cases in which immediate treatment was proposed but the patient did not return for treatment; biopsy failure: cases in which a worse lesion was detected in the first follow-up visit.

A total of 173 women with a histopathological diagnosis of CIN2 were identified, comprising 127 cases from January 2006 to June 2018 and 46 cases from July 2018 to May 2022 (Fig 1). After applying the exclusion criteria, 68 cases met eligibility criteria. Among these, 18 patients were considered lost to follow-up. Thus, 50 patients were included in the analysis of clinical outcomes and baseline characteristics. Of these, 38/50 (76%) had available p16 and Ki-67 immunohistochemistry, while 12 cases lacked biomarker data due to unavailable paraffin blocks or absence of residual lesion on deeper sections. At the end of the study, 76% (38/50; 95% CI: 64.2–87.8%) of cases showed regression, 12% (6/50; 95% CI: 3.0–21%) showed lesion persistence, and 12% (6/50; 95% CI: 3.0–21%) progressed to CIN 3 (5/6) or carcinoma (1/6).

The case demonstrating progression to carcinoma was identified within the retrospective cohort; the patient had discontinued follow-up and returned five years after the initial diagnosis of CIN2.

Table 1 summarizes the baseline characteristics of the included CIN2 cases. In one case, the initial colposcopy report could not be located. Twelve cases did not provide sufficient information on p16 and Ki-67 status due to a lack of paraffin blocks or the absence of CIN2 lesions in the immunohistochemical slides.

**Table 1:** Clinical and laboratory characteristics of Included Patients.

Baselines Characteristics (N = 50)	Mean ± SD^a^ or N (%)	Missing N (%)
Age (N = 50)	30.5 ± 8.8	–
Referral Cytology (N = 50)^b^		–
ASC-US	3 (6.0)	
LSIL	9 (18.0)	
ASC-H	14 (28.0)	
HSIL	24 (48.0)	
Colposcopy (N = 49)		1 (2%)
Minor abnormalities	25 (51.0)	
Major abnormalities	24 (49.0)	
p16 status (N = 38)		12 (24%)
Negative	7 (18.4)	
Positive^c^	31 (81.6)	
Ki-67 status (N = 38)		12 (24%)
Ki-67 Negative	34 (89.5)	
Ki-67 Positive^d^	4 (10.5)	

^a^SD: Standard Deviation; ^b^ASC-US: atypical squamous cells of undetermined significance, LSIL: low-grade squamous intraepithelial lesion, ASC-H: atypical squamous cells of undetermined significance cannot rule out high grade lesion, HSIL: high-grade squamous intraepithelial lesion; ^c^p16 staining was considered positive if it was strong and diffuse in at least one-third of the epithelium; ^d^Ki-67 positivity was defined as staining in ≥50% of cells.

The mean age of the study patients was 30.5 years, ranging from 17.7 to 48.9 years. Most patients were referred to colposcopy with a previous cytology result of HSIL (48%, 24/50), and 51% (25/49) of those cases had minor abnormal colposcopic findings. Immunohistochemical staining of p16 was considered positive in 81.6% (31/38) of cases, and Ki-67 was negative in 89.5% (34/38) of CIN2 biopsies analyzed.

The median time between biopsy and outcome determination was 11.3 months, ranging from 3.7 to 60.2 months of follow-up. During the follow-up period, 38 (76%) patients showed regression, 6 (12%) showed persistence of the lesion, and 6 (12%) progressed to CIN 3 (5) or carcinoma (1). In regressor cases, the median clinical follow-up time was 9.8 months (4.2–32 months); for persistent cases, 12.6 months (3.7–28 months); and for progressing cases, 39.6 months (29.4–60.2 months).

Among women with regression outcome, 79% (30/38) showed complete regression, and 21% (8/38) showed partial regression (regression up to CIN 1 or LSIL). For analysis, complete or partial regression was considered together.

**Fig 2:**
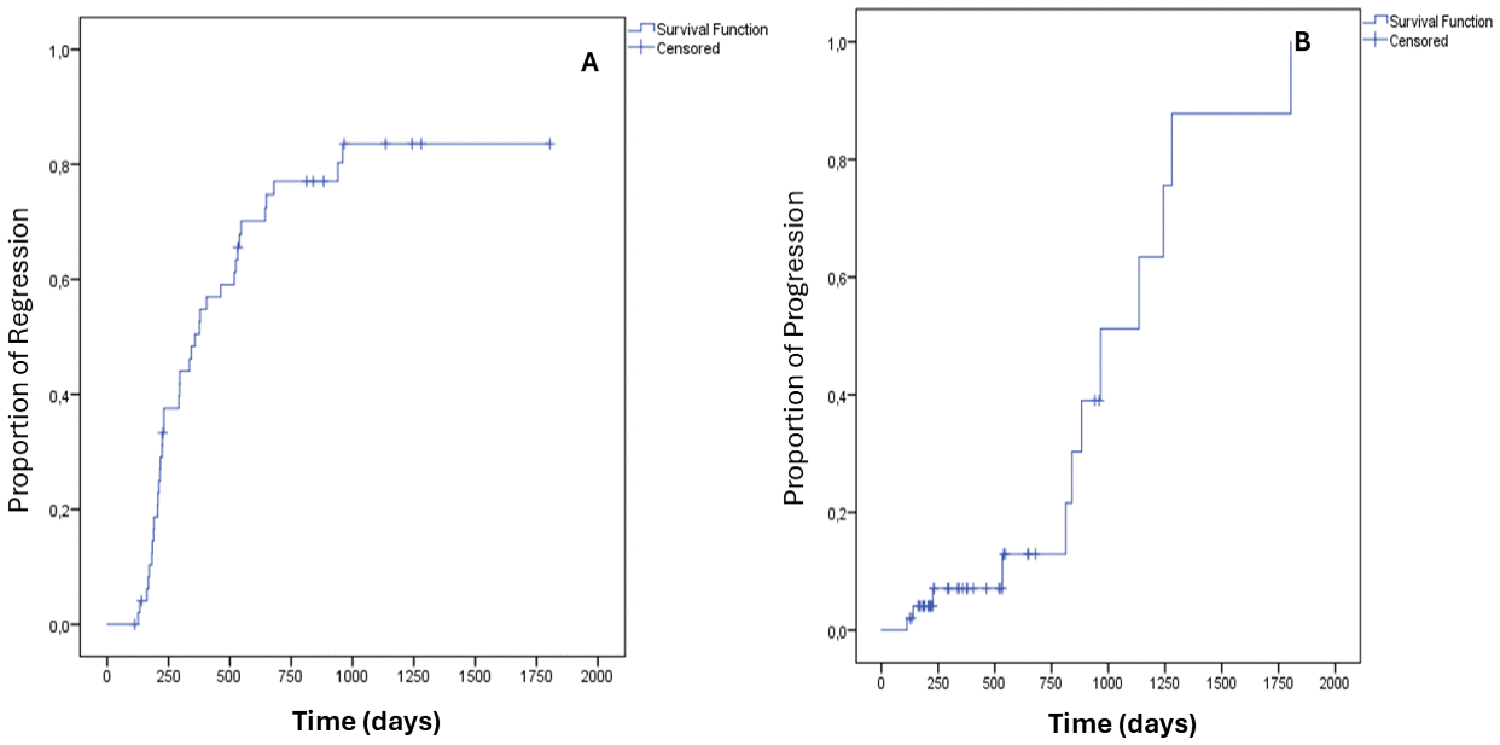
Kaplan-Meier Curves (1-survival): (A) Proportion of regression over time, (B) Proportion of progression over time. Time: time in days since the biopsy. Cross marks (+) indicate censored observations (women who had not yet regressed (A) or progressed (B) or were lost to observation at the time of censoring). Median time to regression = 357 days (SE = 61.4 days; 95% CI: 236.6–477.4 days); median time to progression = 966 days (SE = 159.1 days; 95% CI: 654.1–1277.9 days).

The Kaplan–Meier curves (Fig 2) demonstrate distinct temporal patterns for CIN2 regression and progression. Most regression events occurred early in follow-up. Kaplan-Meier analysis showed that the median time to lesion regression was 357 days (standard error [SE] = 61.4 days; 95% CI: 236.6–477.4 days). In contrast, progression events accumulated more slowly and tended to appear later: Kaplan–Meier analysis showed a median time to progression of 966 days (SE = 159.1 days; 95% CI: 654.1–1277.9 days).

Table 2 presents the bivariate analysis of baseline characteristics and regression outcome. None of the evaluated variables showed a statistically significant association with regression. Still, the highest post-hoc statistical power observed was 24%, corresponding to the association between colposcopic findings and clinical outcomes.

**Table 2:** Bivariate Analysis: association of baseline characteristics and outcomes.

Variable	Outcome		OR (95% CI)	p-value
	Regression (≤CIN1^a^)	No regression (≥CIN2^b^)		
Age (mean ± SD) (N = 50)	30.4 **±** 9.3	30.7 **±** 7.3		
Age < 30 years	15 (93.8%)	1(6.3%)	1.40 (0.38-5.20)	0.614^c^
Age ≥ 30 years	19 (79.2%)	5 (20.8%)	ref	
Referral Cytology (N = 50)				
ASC-US/LSIL	10 (83.3%)	2 (16.7%)	1.78 (0.33-9.59)	0.700^c^
HSIL/ASC-H	28 (73.7%)	10 (26.3%)	ref	
Colposcopy (N = 49)				
Minor abnormalities	19 (76.0%)	6 (24.0%)	1.05 (0.29-3.88)	0.935^d^
Major abnormalities	18 (75.0%)	6 (25.0%)	ref	
p16 status ^e^ (N = 38)				
Negative	5 (71.4%)	2 (28.6%)	1.02 (0.17-6.27)	1.000^c^
Positive	22 (71.0%)	9 (29.0%)	ref	
Ki-67 status ^f^ (N = 38)				
Negative	23 (67.7%)	11 (32.3%)	Not estimable	0.302^c^
Positive	4 (100%)	0 (0.0%)	ref	

^a^ ≤ CIN 1: Negative and CIN 1 (regression); ^b^ ≥ CIN2: CIN2, CIN 3, and carcinoma (no regression); ^c^Fisher`s Exact test; ^d^Chi-Square test (χ²); ^e^strong, diffuse staining in at least one-third of the epithelium; ^f^ staining observed in 50% or more of the cells. Values represent row percentages unless otherwise specified; all observed post-hoc statistical power was ≤ 24% (colposcopic findings and outcomes).

Sensitivity analyses showed no significant differences in the distribution of outcomes between retrospectively identified cases and prospectively enrolled patients (χ² = 3.986, *p* = 0.1363). Similarly, no differences were observed in the distribution of biomarker status across data collection modes (Fisher’s exact test, p16 p = 0.41; Ki-67 p = 0.29) (S1 Dataset).

## Discussion

This cohort study evaluated the natural history of biopsy-confirmed CIN2 managed conservatively in a middle-income country public healthcare setting, describing time to regression and progression, and exploring whether p16, Ki-67, age, referral cytology, or colposcopic findings were associated with regression. We found that 76% (38/50) of CIN2 patients experienced lesion regression. None of the baseline characteristics analyzed – including p16 status, Ki-67 status, age, referral cytology, or colposcopy findings – showed a statistically significant association with regression in bivariate analysis. Given the limited number of events, no multivariable time-to-event regression analyses were performed.

Previous studies have reported that all progressive cases showed positive p16 staining, as per the LAST criteria [31,35]. Another study reported that 80% of cases that progressed were p16 positive [24]. Some studies have found a statistically significant difference between p16 immunohistochemical staining classification and the evaluated outcomes [24,31,35]. In our cohort, 81.6% (31/38) were p16-positive, and 71% of these cases experienced regression.

There was no statistically significant association between p16 status and regression; however, our study lacked sufficient power to rule out such an association. Our study included only CIN2 biopsy-confirmed cases, as confirmed by at least two pathologists. Another cohort study, involving 443 women, found that when focusing solely on expert-confirmed CIN2 cases, the risk of regression did not vary based on p16 status [36].

Similarly, Ki-67 status was not associated with regression. Again, our study lacked sufficient power to demonstrate that no such association exists conclusively. The high percentage (89%) of CIN2 “negative” for Ki-67 was unexpected and could partly be explained by the high (≥50% of stained cells) cut-off applied. This cut-off was chosen because it had been linked to progression in a previous study [24]. Progression cases had a mean labeling index of 25.98% (range 13–39%), suggesting that our higher cut-off may partly explain the lack of association. Variability in sensitivity among Ki-67 antibody clones has also been documented in other settings [37]. Other authors using different cut-offs have reported significant associations [31]. Our results, therefore, do not diminish the potential value of p16 and Ki-67 as biomarkers; instead, they reflect limitations related to cut-off selection and sample size in the present study. The need for larger, standardized studies to confirm the predictive utility of these biomarkers—particularly in untreated CIN2 cohorts—has also been emphasized by a recent systematic review on predictive biomarkers of clinical evolution in conservatively managed patients [38].

Our study, as well as others [31,39], found no significant difference in patient outcomes by age. However, this observation is likely influenced by limited statistical power to detect age-specific effects. Therefore, the lack of statistical significance does not exclude a potential biological role of age in the evolution of CIN2. Rather, it underscores the need for prospective studies assessing conservative management of CIN2, regardless of age, provided adequate follow-up is ensured.

The median time to regression events was nearly 1 year (357 days; 95% CI: 236.6–477.4 days), but among cases that progressed, this period was about 3 times longer (966 days; SE: 159.1; 95% CI: 654.1–1277.9). The Kaplan-Meier curves demonstrated that regression is typically an early event, whereas progression tends to occur later. The probability of regression rose steeply within the first two to three years. It plateaued thereafter, while the cumulative probability of progression remained low during the first two years and increased gradually thereafter, with all progression events occurring after a two-year follow-up. This finding is partly due to the predetermined treatment time in persistent cases.

These data suggest that a 24-month period of active surveillance is an appropriate and safe timeframe for the conservative management of CIN2, provided patients comply with follow-up protocols. Following this period, cases that do not regress should be re-evaluated to determine whether continued surveillance or treatment is necessary. This interpretation aligns with prior studies reporting regression rates of 64%−74% after 2 years of surveillance [39,40] and is consistent with the meta-analysis by Tainio et al. [5], which supports conservative management.

Despite some previous studies reporting no carcinoma cases in patients with CIN2 biopsies [7,9], our retrospective data identified one case (2%) of progression to carcinoma after five years from the initial diagnosis of CIN2. The patient had abandoned follow-up and returned three years later. Another cohort study reported six cases of progression to carcinoma (0.2%) [41]. Their cases with this outcome had persistent lesions with high-grade cytology and/or previous high-risk HPV. Half of these patients were diagnosed with cancer and progressed after a 2- to 3-year interval in follow-up. A recent Danish cohort population-based study reported an increased long-term risk of cervical cancer for women undergoing active surveillance for CIN2 compared to those who received immediate surgical excision [42]. These findings highlight the importance of continuing clinical follow-up for some time, even after regression, given the risk of lesion recurrence.

Management of CIN2 depends on clinical factors and patient preferences, especially regarding obstetric risks. While excisional procedures are known to increase obstetric complications [12], a recent cohort study showed similar preterm birth rates for active surveillance and immediate excision. Delayed excision, however, raised the risk of preterm birth by 30% compared to immediate surgery [43].

However, the choice of clinical management in this group of patients remains a challenge, as histopathological results are still used as the gold standard. It is widely documented in the literature that the interpretation and reproducibility of biopsies are limited. In addition to low interobserver agreement, especially in cases of CIN2, this diagnostic category is also notoriously heterogeneous, including transient HPV lesions and lesions with progressive potential. For these reasons, interest in criteria or biomarkers that can predict and differentiate truly progressive lesions from regressive ones is increasing [39].

Our study sought to minimize low interobserver reproducibility by including only CIN2 biopsy cases evaluated by at least two pathologists. Additionally, all cases were reassessed by an experienced colposcopist to determine outcomes, especially whether cases considered to be progression were truly CIN2 lesions that had progressed, or higher-grade lesions (CIN 3 and carcinoma) present on the cervix but not represented in the initial biopsy. These findings most likely reflect sampling error or underrepresentation of a higher-grade lesion at the time of biopsy (biopsy failure), rather than actual biological progression over such a short interval. These cases were excluded to avoid misclassification and to ensure that only actual CIN2 cases under conservative management were included in the analysis.

Also, cases diagnosed by excisional biopsy were excluded to avoid outcome misclassification, as these lesions would be incorrectly classified as “regression” despite being physically removed rather than undergoing spontaneous biological regression. Inclusion of such cases would artificially inflate regression rates and distort the natural history under conservative management.

However, our study has significant limitations. The limited sample size reduced statistical power, particularly for analyses exploring associations between immunohistochemical markers and lesion regression. Therefore, non-significant findings should not be interpreted as an absence of association. A larger, multicenter dataset will be necessary to evaluate predictive factors with adequate precision.

This study included both retrospectively identified and prospectively enrolled patients, which may have introduced some heterogeneity related to data collection. However, all analyses were conducted using the same predefined protocols, and sensitivity analyses showed no significant differences in the distribution of outcomes or biomarker status between cohorts, suggesting that the bidirectional design is unlikely to have introduced substantial bias into the results.

p16 and Ki-67 immunohistochemistry were not performed routinely in clinical practice and were assessed retrospectively for research purposes, depending on the availability and adequacy of archived biopsy material. Consequently, missing biomarker data were unlikely to be missing at random. Importantly, however, these missing data were not associated with clinical outcomes, suggesting a low risk of bias. In addition, immunohistochemical results were not used to guide clinical management and were evaluated solely to explore potential associations with clinical outcomes.

The study reflects the Brazilian public healthcare system (SUS), in which cervical screening is opportunistic; generalizability to other settings with different screening infrastructures should be approached with caution.

These limitations, particularly the small sample size, mean that our inability to detect predictive value for baseline characteristics does not rule out their potential importance. Despite the limited statistical power, this study provides a well-characterized cohort of women with biopsy-confirmed CIN2 cases managed conservatively under a standardized follow-up protocol. The prospective assessment of outcomes and the detailed clinical, histopathological, and immunohistochemical characterization contribute valuable real-world data from a public healthcare setting in a middle-income country. Importantly, by making these data openly available, this study aims to facilitate support for future pooled analyses and multicenter collaborations, in which larger sample sizes may allow more robust evaluation of potential predictive factors in CIN2 management.

## Conclusion

In this study, CIN2 regression occurred in 76% of the included cases, with median times of approximately 12 months for regression and 32 months for progression.

No statistically significant associations between baseline characteristics, including biomarkers, and lesion regression were observed in this cohort; however, the exploratory nature of the analysis, limited sample size, and substantial censoring preclude definitive conclusions.

## Supporting information

S1 Dataset An Excel spreadsheet containing detailed information on all 50 CIN2 cases included in the study. (XLS)
